# Midazolam increases preload dependency during endotoxic shock in rabbits by affecting venous vascular tone

**DOI:** 10.1186/s13613-018-0403-9

**Published:** 2018-05-02

**Authors:** Jianxiao Chen, Tao Yu, Federico Longhini, Xiwen Zhang, Songqiao Liu, Ling Liu, Yi Yang, Haibo Qiu

**Affiliations:** 1 Department of Critical Care Medicine, Zhongda Hospital, School of Medicine, Southeast University, 87 Dingjiaqiao Road, Nanjing, 210009 People’s Republic of China; 2 Anesthesia and Intensive Care, Department of Translational Medicine, Eastern Piedmont University “A. Avogadro”, Novara, Italy

**Keywords:** Midazolam, Preload dependency, Vascular resistance, Endotoxic shock, Mean systemic filling pressure

## Abstract

**Background:**

Septic patients often require sedation in intensive care unit, and midazolam is one of the most frequently used sedatives among them. But the interaction between midazolam and septic shock is not known. The aim of this study is to investigate the effects of midazolam on preload dependency in an endotoxic shock model by evaluating systemic vascular tone and cardiac function.

**Methods:**

Eighteen rabbits were randomly divided into three groups: Control group, MID1 group and MID2 group. Rabbits underwent ketamine anaesthesia and mechanical ventilation, and haemodynamic assessments were recorded in three groups (T0). Endotoxic shock was induced by lipopolysaccharide intravenously, and fluid resuscitation and norepinephrine were administered to obtain the baseline mean arterial pressure (MAP) (T1). Rabbits received equivalent normal saline (Control) and two consecutive dosages of midazolam: 0.3 mg kg^−1^ h^−1^ (MID1) and 3 mg kg^−1^ h^−1^ (MID2) (T2). Rabbits received another round of fluid challenge and norepinephrine infusion to return the MAP to normal (T3).

**Results:**

No significant differences in haemodynamic parameters were observed in three groups at T0, T1 or T3. Midazolam infusion significantly increased pulse pressure variation (PPV) and stroke volume variation (SVV) compared to the values in Control group, and MAP, central venous pressure (CVP), mean systemic filling pressure (Pmsf) and cardiac output (CO) decreased at T2. Same effects were observed with increasing doses of midazolam, and resistance for venous return (Rvr) decreased (MID1 vs. MID2) at T2. PPV and SVV increased significantly at T2 compared to the values at T1. MAP, CVP, Pmsf and CO decreased in MID1 and MID2 groups. Rvr also decreased in MID2 group (T2 vs. T1). Midazolam did not affect cardiac function index, systemic vascular resistance or artery resistance (T2 vs. T1).

**Conclusions:**

Midazolam administration promoted preload dependency in septic shock models via decreased venous vascular tone without affecting cardiac function.

## Background

Septic shock is a deleterious systemic host response to infection characterized by hypotension that is not reversed with fluids alone. Septic shock is a common reason for admission to the intensive care unit (ICU) [1]. The response to fluid challenge is complicated by cardiovascular physiology, but it plays an important role in the resuscitation of sepsis patients [2]. However, fluid responsiveness only occurs in half of critically ill patients, including patients with sepsis [3]. Fluid resuscitation is a mainstay of early treatment, but the deleterious effects of excessive fluid administration that lead to tissue oedema are becoming clearer.

Patients with septic shock generally require mechanical ventilation, which makes the use of sedative drugs almost imperative to reduce anxiety and agitation and facilitate care. Benzodiazepines (e.g. midazolam) are commonly used to sedate patients in the ICU, and a recent survey demonstrated that midazolam remains widely used [4]. Benzodiazepines inhibit the activity of the autonomic nervous system [5, 6]. Midazolam attenuates the release of catecholamines in vivo and induces vasoplegia, which contributes to the resulting haemodynamic changes [7, 8].

Norepinephrine, an α1-agonist drug, is recommended as a first-line vasopressor [9]. Norepinephrine reduces the preload dependency via exertion on arterial and venous tone to increase systemic arterial resistance, primarily by recruiting blood from the large venous unstressed volume [10]. Our previous work demonstrated that propofol and dexmedetomidine increased preload dependency in an endotoxic shock model after fluid resuscitation during norepinephrine infusion, and the mechanism primarily relied on the systemic vascular system and cardiac function [11]. Few studies have reported the haemodynamic effects of midazolam infusion in endotoxic shock models during norepinephrine infusion.

In the present experimental, randomized study, we investigated the effects of midazolam on preload dependency in rabbits subjected to endotoxic shock with norepinephrine infusion by evaluating the systemic vascular system and cardiac function.

## Methods

### Ethics statement

New Zealand white rabbits (3.26 ± 0.14 kg body weight) were obtained from the animal centre of Southeast University and housed in a pathogen-free environment on a 12-h light/dark cycle with free food and water access for at least 5 days prior to experimentation. All animals received care according to the Helsinki convention for the use and care of animals, the “Principles of Laboratory Animal Care” formulated by the National Society for Medical Research and the “Guide for the Care and Use of Laboratory Animals” by the China National Academy of Sciences. The Academic Ethical Committee of Southeast University Medical School, Nanjing, China, approved the study protocol, which has been described previously [11].

### Animal preparation

Rabbits received an intramuscular injection of ketamine (20 mg kg^−1^) and atropine (0.15 mg kg^−1^), which was used to reduce mucosal secretion in the airways. A marginal ear vein was cannulated to guarantee intravenous anaesthesia using ketamine (3 mg kg^−1^ h^−1^) during the entire study protocol, as previously described [11, 12]. A tracheotomy was performed after local anaesthesia with lidocaine, and a 3.5–4-mm-inner-diameter endotracheal tube was placed. Rabbits were ventilated using a Servo-I with proper software for neonatal and paediatric ventilation (Maquet Critical Care, Solna, Sweden). Tracheal cannulation was used to better adapt the rabbits to controlled mechanical ventilation and avoid spontaneous breathing. A continuous infusion of vecuronium (0.05 mg kg^−1^ h^−1^) was administered for neuromuscular block, and an adjunctive bolus of 0.5–1 mg was added to optimize the animal curarization if needed.

Rabbits were ventilated via volume control ventilation with the following settings: zero end-expiratory pressure, a tidal volume equal to 8 mL kg^−1^, an initial respiratory rate equal to 40 breath min^−1^ (modified according to the carbon dioxide partial pressure targeted to the physiological range) and an inspired fraction of oxygen of 60%. Arterial blood was sampled for gas analysis to adjust the ventilator setting in case of respiratory acidosis prior to endotoxic shock induction. The right internal jugular vein and femoral artery were surgically isolated, and a central vein catheter was placed to infuse fluids and drugs. A dedicated arterial thermodilution catheter (4 Fr, 8 cm Pulsiocath PV2014L16; Pulsion Medical Systems, Munich, Germany) was inserted to acquire the haemodynamic measurements [12]. Lactate Ringer’s solution (4 mL kg^−1^ h^−1^) was infused in the central vein catheter, and 2 mL h^−1^ of normal saline with 4 IU mL^−1^ of heparin was infused through the arterial line. Blood temperature was monitored and maintained between 38 and 39 °C via a warming lamp.

An intravenous infusion over 30 s of 0.5 mg kg^−1^ *E. coli* LPS (O55:B5; Sigma Chem. Co., St. Louis, MO, USA) was used to induce endotoxic shock, which was confirmed by a 25% decrease in mean arterial pressure (MAP) [13]. Fluid resuscitation (20 mL, intravenous bolus) was administered to all endotoxic rabbits, and 50 mL kg^−1^ fluid was injected for another 2 h to maintain blood pressure. Norepinephrine infusion was initiated, and the dose was titrated to maintain MAP at baseline values and remain constant throughout the entire protocol. The haemodynamic variables were allowed to stabilize, which was assessed as a variation of MAP < 10% over a 30-min period [14].

### Experimental protocol

**Fig. 1:**
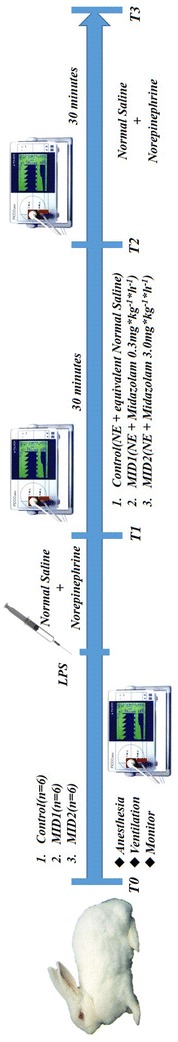
Flowchart of the experiment protocol. After animal preparation (T0), an intravenous infusion of 0.5 mg kg^−1^ of LPS over 30 s was used to induce the endotoxic status, which was confirmed by a 25% decrease in mean arterial pressure. All animals received fluid resuscitation of 20 mL normal saline intravenously and then 50 mL kg^−1^ normal saline for another 2 h to maintain blood pressure. Norepinephrine infusion was initiated after fluid resuscitation and titrated to maintain the baseline blood pressure (T1). Midazolam was intravenously infused at doses of 0.3 or 3 mg kg^−1^ h^−1^ (T2). Equivalent normal saline was administered to rabbits in the Control group. A second round of fluid resuscitation and norepinephrine infusion was initiated to return the blood pressure back to normal (T3)

Rabbits were randomly divided into three groups (*n* = 6 in each group): Control group, MID1 group and MID2 group. Figure 1 shows the flowchart of the study protocol. Endotoxic shock was initiated after animal preparation (T0), and the following fluid resuscitation and norepinephrine infusions were administered to all three groups. Haemodynamic measurements were obtained after stabilization (T1). Rabbits received two consecutive dosages of midazolam for 30 min: 0.3 mg kg^−1^ h^−1^ (MID1 group) and 3 mg kg^−1^ h^−1^ (MID2 group). Rabbits in the Control group received equivalent doses of normal saline. Haemodynamic measurements were performed at the end of the 30 min trial, and the data were recorded (T2). Rabbits received another round of fluid challenge and norepinephrine infusion to return the MAP to normal (T3).

### Haemodynamic measurements

Heart rate (HR), systolic blood pressure (SBP), diastolic blood pressure (DBP), MAP and central vein pressure (CVP) were continuously monitored and recorded. Haemodynamic measurements were performed using a dedicated indwelling arterial catheter for the PiCCO Plus device (Pulsion Medical Systems, Munich, Germany).

Proper calibration of the PiCCO Plus for pulse contour analysis was performed at each measurement time point using two 3-mL bolus injections of 4 °C normal saline. A third calibrating injection was performed if the first two values differed by more than 10%.

Stroke volume (SV), cardiac output (CO) and global end-diastolic volume (GEDV) were acquired via transpulmonary dilution [11, 15]. Pulse pressure variation (PPV) and stroke volume variation (SVV) were calculated for preload dependency.

Systemic vascular resistance (Rsys), mean systemic filling pressure (Pmsf), resistance to venous return (Rvr) and arterial resistance (Ra) were calculated as previously described [14, 16]. Briefly, end-inspiratory occlusions were performed at different levels of positive end-expiratory pressure (PEEP), and the extreme values of CO and CVP were recorded simultaneously. Each pair of measurements was plotted on a graph connecting CO (*Y*-axis) and CVP (*X*-axis), and the regression line was computed using the least-squares method in Microsoft Excel. Pmsf was estimated as the pressure that corresponded to the X-intercept of the regression line, and resistance to the venous return was calculated as the inverse of the slope of the line. Rsys was calculated as (MAP-CVP)/CI. Ra was estimated as (MAP-Pmsf)/CI, and Rvr was calculated as (Pmsf-CVP)/CI.

The Cardiac Function Index (CFI) was calculated as the ratio of CO × 1000 to GEDV, and it was recorded as an estimate of ventricular systolic function [6, 11, 14, 17]. The ventilator settings, anaesthesia and vasoactive drugs were not modified during the study protocol.

### Blood gas measurements

Blood gas measurements were obtained from the arterial and venous catheters at T0, T1, T2 and T3 to measure pH, the partial pressure of carbon dioxide (PCO_2_), the ratio of alveolar oxygen partial pressure to the fraction of inspiration O_2_ (P/F), lactic acid (Lac), haemoglobin (Hb), bicarbonate (HCO_3_^−^) and oxygen saturation of mixed venous blood (SvO_2_).

### Statistics

Data were analysed using SPSS 19.0 for Windows (SPSS Inc., Chicago, IL, USA) and GraphPad Prism 7 for Windows (GraphPad Prism Software Inc., La Jolla, CA, USA). We computed the descriptive statistics for all study variables. We used the Kolmogorov–Smirnov test and stratified the distribution plots to verify the distribution normality of the continuous variables. Data that were normally distributed are presented as the mean ± standard deviation (SD), and non-normally distributed data are presented as medians (interquartile, IQ). We assessed differences in the distribution normality of the continuous variables using one-way analyses of variances followed by Bonferroni corrections for multiple comparisons. We used the Mann–Whitney *U* test to evaluate non-normally distributed data. *p *< 0.05 was considered statistically significant for all analyses (Table 1).

**Table 1:** Haemodynamic values for each group at baseline and after fluid resuscitation and norepinephrine infusion

	T0			T1			T2			T3		
	Control	MID1	MID2	Control	MID1	MID2	Control	MID1	MID2	Control	MID1	MID2
HR (/min)	220 [213–221]	221 [219–229]	222 [219–226]	192 [190–192]△	201 [191–204]△	191 [190–194]△	188 [185–191]△	219 [215–228]*&	218 [211–224]*&	193 [191–195]△	202 [189–203]△^	192 [191–194]△^
SBP (mmHg)	131 [130–134]	130 [129–130]	130 [126–135]	142 [136–150]△	148 [141–151]△	146 [141–152]△	143 [137–150]△	129 [126–133]*&	108 [107–108]*#△&	135 [131–141]	137 [135–138]&	142 [135–147]△^
DBP (mmHg)	78 [76–80]	79 [75–80]	77 [75–80]	84 [81–85]	86 [82–88]	88 [82–89]	80 [79–84]	71 [70–77]&	57 [56–60]*#△&	79 [78–82]	80 [76–80]^	77 [76–78]&^
MAP (mmHg)	96 [92–98]	94 [93–96]	96 [92–97]	105 [98–108]△	104 [101–108]△	106 [104–110]△	103 [95–107]△	90 [87–95]*&	74 [72–76]*#△&	99 [96–103]	98 [97–99]&^	97 [96–99]&^
CVP (mmHg)	0 [0–1]	1 [0–1]	0 [0–0]	4 [3, 4]△	4 [3–5]△	2 [2–4]△	4 [3–4]△	3 [3–3]△&	1 [1–2]*#△&	4 [3–4]△	5 [4–5]△^	3 [2–4]△^
CO (L/min)	0.70 [0.68–0.70]	0.71 [0.69–0.72]	0.79 [0.77–0.81]	0.97 [0.95–0.98]△	0.90 [0.89–0.96]△	0.96 [0.95–1.01]△	0.97 [0.95–1.00]△	0.83 [0.82–0.88]*△&	0.74 [0.71–0.76]*&	0.97 [0.95–0.98]△	0.90 [0.89–0.96]△^	0.96 [0.95–1.01]△^
SV (mL)	3.16 [2.97–3.42]	3.19 [3.13–3.40]	3.53 [3.46–3.64]	5.04 [4.97–5.09]△	4.77 [4.48–4.84]△	4.96 [4.82–5.28]△	5.04 [4.91–5.13]△	3.96 [3.76–4.07]*△&	3.42 [3.18–3.67]*&	4.99 [4.93–5.06]△	4.72 [4.46–4.94]△^	4.96 [4.81–5.25]△^
GEDV (mL)	51.73 [50.96,51.95]	52.61 [52.42,53.10]	52.29 [51.96,52.93]	85.10 [79.61,91.04]△	83.68 [80.95,86.80]△	84.04 [77.73,85.20]△	85.60 [79.14,90.79]△	79.88 [74.58,81.53]△&	61.15 [58.22,63.21]*#△&	85.15 [79.36,90.35]△	87.28 [81.01,87.96]△^	84.84 [77.70,87.06]△^
PPV (%)	19 [17–21]	17 [16–21]	18 [17–19]	12 [9–14]△	9 [7–10]△	9 [8–10]△	12 [10–14]△	14 [12–14]△&	18 [17–20]*#&	12 [10–14]△	10 [10–11]△	11 [9–12]△^
SVV (%)	20 [18–22]	19 [17–21]	17 [16–21]	12 [11–13]△	9 [7–10]△	10 [8–10]△	11 [10–12]△	15 [14–16]*△&	19 [18–20]*#&	11 [10–12]△	10 [9–11]△^	10 [8–10]△^
Pmsf (mmHg)	11.38 [10.66–11.74]	11.25 [9.82–12.18]	10.21 [9.84–10.83]	15.10 [14.37–15.33]△	15.25 [12.80–15.90]△	13.66 [13.32–14.44]△	13.96 [12.62–15.19]△	11.99 [11.39–12.33]*&	7.47 [7.12–8.18]*#△&	15.04 [14.43–15.21]△	14.67 [13.05–16.48]△^	13.84 [11.78–14.05]△^
Rsys (mmHg min kg/L)	433.07 [407.43–467.57]	425.39 [415.13–447.27]	391.68 [385.29–396.17]	313.44 [285.22–349.96]△	344.17 [335.74–378.40]△	345.42 [329.23–359.34]△	307.07 [275.76–354.48]△	325.91 [319.91–364.31]△	321.84 [315.46–330.69]△	304.85 [277.58–330.59]△	330.35 [329.47–345.24]△	311.64 [295.34–331.80]△
Ra (mmHg min kg/L)	381.99 [359.36–415.32]	376.87 [371.82–395.45]	351.96 [348.17–354.64]	281.67 [246.52–309.64]△	309.23 [297.37–339.03]△	315.84 [291.55–322.58]△	277.14 [238.35–317.62]△	294.21 [284.26–328.25]△	295.30 [286.45–306.61]△	268.01 [240.22–293.72]△	294.57 [288.36–300.60]△	279.84 [267.47–298.88]△
Rvr (mmHg min kg/L)	50.00 [47.22–52.25]	47.09 [44.35–51.98]	42.41 [38.80–44.46]	37.02 [30.15–39.24]△	39.42 [35.58–39.94]△	37.12 [32.12–39.78]	36.71 [26.93–37.80]△	36.04 [33.01–36.69]△	28.05 [26.93–29.48]#△&	36.80 [29.28–41.45]△	36.90 [29.71–41.42]△	32.08 [30.10–33.84]△
CFI (/min)	13.41 [13.25–13.51]	13.52 [13.22–13.55]	14.89 [14.35–15.52]	11.82 [11.33–12.46]△	10.97 [10.62–11.19]△	12.23 [11.54–12.30]△	11.86 [11.29–12.49]△	10.81 [10.25–11.29]△	12.26 [11.34–12.46]△	11.86 [11.29–12.49]△	10.81 [10.25–11.29]△	12.26 [11.34–12.46]△

The table shows the recorded haemodynamic values expressed as medians (IQR) of animal preparation at baseline (T0), after fluid resuscitation and norepinephrine infusion (T1), after midazolam infusion (T2) and after the second round of fluid resuscitation (T3) in the Control, MID1 and MID2 groups

*HR* heart rate, *SBP* systolic blood pressure, *DBP* diastolic blood pressure, *MAP* mean arterial pressure, *CVP* central venous pressure, *CO* cardiac output, *SV* stroke volume, *GEDV* global end-diastolic volume, *PPV* pulse pressure variation, *SVV* stroke volume variation, *Pmsf* mean systemic filling pressure, *Rsys* systemic vascular resistance, *Ra* resistance for artery, *Rvr* resistance for venous return, *CFI* Cardiac Function Index

*p < 0.05 versus Control, #*p *< 0.05 versus MID1, △*p *< 0.05 versus T0, &*p *< 0.05 versus T1, ^*p *< 0.05 versus T2, *n* = 6

## Results

Eighteen rabbits were anaesthetized for the study protocol. Endotoxic shock was successfully established in all animals, as indicated by a 25% decrease in MAP. Fluid resuscitation and norepinephrine infusion (Table 2) restored MAP to the initial value prior to endotoxic shock. The rabbits received a second fluid challenge and norepinephrine infusion after midazolam infusion to return the MAP to normal. No differences were detected between the Control, MID1 and MID2 groups with respect to the time to achieve endotoxic shock (29.1 ± 6.8, 28.4 ± 7.2 and 29.0 ± 7.0 min, respectively; *p *> 0.05) or the volume of administered fluid during T0–T1 and T1–T2. The volume of administered fluid increased from T2 to T3 between the Control, MID1 and MID2 groups (29.10 ± 1.46, 45.40 ± 1.19 and 65.21 ± 1.16 mL, respectively, *p *< 0.05). No differences were detected between the Control, MID1 and MID2 groups with respect to the norepinephrine infusion rate (5.51 ± 0.23, 5.55 ± 0.21 and 5.56 ± 0.27 mcg kg^−1^ min^−1^, respectively, *p *> 0.05). Blood gases confirmed normal baseline status, and there were no significant differences between T0, T1, T2 or T3 among all three groups (Table 3). No rabbits died.

**Table 2:** Fluid and norepinephrine administration during the experiment

Treatment	T0–T1			T1–T2			T2–T3		
	Control	MID1	MID2	Control	MID1	MID2	Control	MID1	MID2
Saline (mL)	343.33 ± 16.32	350.00 ± 12.64	345.00 ± 13.78	29.10 ± 1.46	29.70 ± 1.13	29.25 ± 1.24	29.10 ± 1.46	45.40 ± 1.19*	65.21 ± 1.16*#
Norepinephrine (mcg kg^−1^ min^−1^)	5.51 ± 0.23	5.55 ± 0.21	5.56 ± 0.27	5.51 ± 0.23	5.55 ± 0.21	5.56 ± 0.27	5.51 ± 0.23	5.55 ± 0.21	5.56 ± 0.27

Data are shown as the mean ± SD

T0: baseline; T1: endotoxic shock after fluid resuscitation and norepinephrine infusion; T2: after the administration of midazolam at 0.3 mg kg^−1^ h^−1^ (MID1) or 3 mg kg^−1^ h^−1^ (MID2); T3: after second round of fluid resuscitation

*p < 0.05 versus control, #*p *< 0.05 versus MID1

**Table 3:** Analysis of blood gas with increasing midazolam infusion rates

	T0			T1			T2			T3		
	Control	MID1	MID2	Control	MID1	MID2	Control	MID1	MID2	Control	MID1	MID2
pHa	7.30 ± 0.09	7.34 ± 0.03	7.27 ± 0.07	7.23 ± 0.05	7.24 ± 0.03	7.38 ± 0.06	7.23 ± 0.08	7.22 ± 0.05	7.24 ± 0.07	7.25 ± 0.06	7.24 ± 0.03	7.32 ± 0.06
PCO_2_ (mmHg)	33.78 ± 9.57	33.93 ± 5.07	33.70 ± 6.14	35.63 ± 7.46	33.91 ± 4.92	34.12 ± 5.68	33.71 ± 6.14	34.10 ± 5.07	36.71 ± 6.02	33.81 ± 5.98	33.98 ± 5.12	35.12 ± 5.82
P/F (mmHg)	339.65 ± 71.59	308.55 ± 35.01	348.02 ± 64.01	299.75 ± 69.52	296.15 ± 59.58	297.68 ± 67.35	292.51 ± 50.51	298.30 ± 21.10	290.51 ± 38.62	296.62 ± 48.91	298.50 ± 22.30	298.36 ± 35.72
Lac (mmol/L)	2.90 ± 0.69	2.90 ± 0.54	2.45 ± 0.97	2.91 ± 1.31	2.93 ± 1.35	3.12 ± 1.02	2.2 ± 0.98	2.3 ± 0.85	2.91 ± 1.01	2.1 ± 0.87	2.2 ± 0.75	2.6 ± 0.98
Hb (g/dL)	8.5 ± 0.5	8.5 ± 0.6	8.5 ± 0.6	8.3 ± 0.5	8.6 ± 0.7	8.3 ± 0.8	8.3 ± 0.5	8.4 ± 0.3	8.5 ± 0.4	8.6 ± 0.4	8.2 ± 0.5	8.5 ± 0.4
HCO_3_^−^ (mmol/L)	16.2 ± 2.4	16.1 ± 1.6	16.7 ± 3.3	15.79 ± 1.9	16.25 ± 2.3	16.35 ± 1.8	16.68 ± 3.42	16.31 ± 2.31	16.31 ± 2.52	17.08 ± 3.06	16.52 ± 2.11	17.31 ± 1.98
SvO_2_ (%)	88.13 ± 2.93	88.18 ± 2.98	85.68 ± 4.27	85.46 ± 3.96	85.17 ± 3.12	86.35 ± 3.03	85.67 ± 4.12	85.28 ± 3.12	85.69 ± 3.06	86.28 ± 4.35	86.28 ± 4.84	85.39 ± 2.98

Data are shown as the mean ± SD

T0: baseline; T1: endotoxic shock after the fluid resuscitation and norepinephrine infusion; T2: after the administration of midazolam at 0.3 mg kg^−1^ h^−1^ (MID1) or 3 mg kg^−1^ h^−1^ (MID2); T3: after the second round of fluid resuscitation

pHa, pH of artery; PCO_2_, partial pressure of carbon dioxide; P/F, alveolar oxygen partial pressure/fraction of inspiration O_2_; Lac, lactic acid; Hb, haemoglobin; HCO_3_^−^, bicarbonate; SvO_2_, oxygen saturation of mixed venous blood; SD, standard deviation

There was no significant difference between Control, MID1 and MID2 groups at T0, T1, T2 or T3, respectively

Table 1 shows the effects of midazolam on haemodynamics. No differences between the Control, MID1 and MID2 groups were observed at T0, which demonstrates that the study population was homogeneous prior to the initiation of the sedative infusion (*p *> 0.05).

### Effects of midazolam on preload dependency

**Fig. 2:**
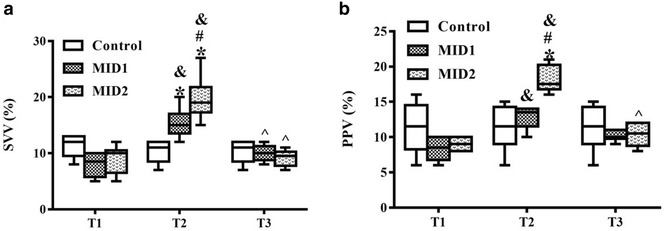
Midazolam increased preload dependency of endotoxic shock rabbits. **a** The effects of midazolam on pulse pressure variation between the Control, MID1 and MID2 groups at T1, T2 and T3. **b** The effects of midazolam on stroke volume variation between the Control, MID1 and MID2 groups at T1, T2 and T3. *PPV* pulse pressure variation, *SVV* stroke volume variation; *p < 0.05 versus Control, #*p *< 0.05 versus MID1, &*p *< 0.05 versus T1, ^*p *< 0.05 versus T2, *n* = 6

Table 1 shows that no differences in PPV or SVV were observed between groups at T0. No differences in PPV or SVV were observed after modelling and resuscitation between the three groups, which demonstrates that all rabbits were without fluid responsiveness at T1. Midazolam administration significantly increased PPV in the MID2 group at T2 (*p *< 0.05), and it significantly increased SVV in the MID1 and MID2 groups (*p *< 0.05) compared to that in the Control group (Fig. 2). SVV in the MID2 group was significantly higher than that in the MID1 group at T2 (*p *< 0.05) (Fig. 1b). No differences were detected in PPV or SVV between groups at T3 (*p *> 0.05).

PPV and SVV decreased from T0 to T1 in all groups (*p *< 0.05) but increased significantly in the MID1 and MID2 groups at T2 compared to the values at T1 (*p *< 0.05). PPV and SVV decreased in the MID1 and MID2 groups from T2 to T3 (*p *< 0.05), but no differences were detected in the Control group (Table 1 and Fig. 2).

### Effects of midazolam on haemodynamic parameters

As shown in Table 1, there were no significant differences in the haemodynamic parameters among the three groups at T0, T1 and T3. However, MAP and Pmsf decreased significantly in the MID1 and MID2 groups (*p *< 0.05), and CVP and CO decreased in the MID2 group compared to the values in the Control group at T2 (*p *< 0.05) (Table 1). MAP, CVP and Pmsf in the MID2 group were significantly lower than the values in the MID1 group at T2 (*p *< 0.05) (Table 1).

**Fig. 3:**
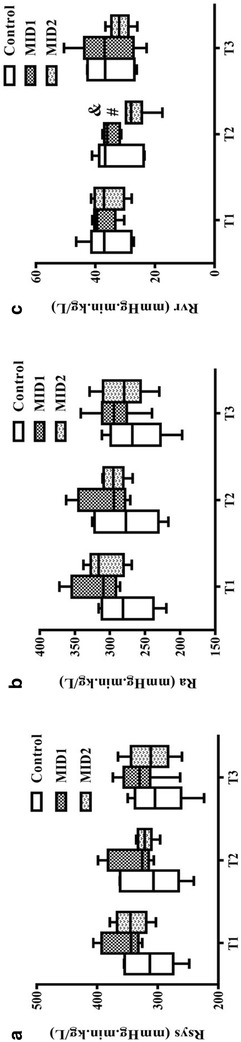
The effects of midazolam on vascular resistance between the Control, MID1 and MID2 groups at T1, T2 and T3. **a** The effects of midazolam on systemic vascular resistance between the Control, MID1 and MID2 groups at T1, T2 and T3. **b** The effects of midazolam on artery resistance between the Control, MID1 and MID2 groups at T1, T2 and T3. **c** The effects of midazolam on resistance for venous return between the Control, MID1 and MID2 groups at T1, T2 and T3. *Rsys* systemic vascular resistance, *Ra* resistance for artery, *Rvr* resistance for venous return; #*p *< 0.05 versus MID1, &*p *< 0.05 versus T1, *n* = 6

Midazolam dosage did not affect Rsys or Ra at T2 or T3, but Rvr deceased significantly in the MID2 group compared to that in the Control group at T2 and T3 (*p *< 0.05) (Fig. 3). There were no differences in CFI between groups at T2 or T3 (*p *> 0.05).

MAP, CVP, Pmsf, CO and SV increased from T0 to T1 and T2 to T3 in all three groups (*p *< 0.05). Rsys, Ra, Rvr, HR and CFI decreased significantly from T0 to T1 (*p *< 0.05) (Table 1). MAP, CVP, Pmsf and CO decreased in the MID1 and MID2 groups at T2 compared to the values at T1 (*p *< 0.05), and the opposite results occurred at T3 compared to the values at T2 (Table 1). Rvr only decreased in the MID2 group at T2 (*p *< 0.05). No differences were detected in Rvr from T2 to T3 (Fig. 3, *p *> 0.05).

## Discussion

To our knowledge, this study is the first to investigate the effects of two midazolam doses on haemodynamics in an endotoxic shock model during norepinephrine infusion. The main results can be summarized as follows: (1) midazolam increased the preload dependency, reduced Pmsf, CVP, GEDI and Pvr and affected the SV and CO despite the increase in HR; (2) no effects on cardiac contractile function as expressed by the CFI were observed. Thus, midazolam primarily affects the heart by increasing venous capacitance.

**Fig. 4:**
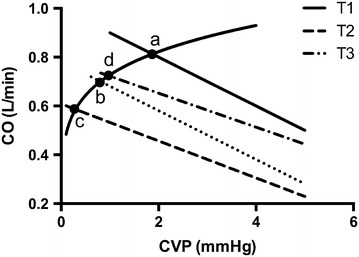
Schematic diagram of the effects of midazolam. Venous return curve and cardiac output curve constructed from the average values of central venous pressure, mean systemic filling pressure and cardiac output after resuscitation and midazolam infusion. The dots are the values derived from Table 1. (a) The working point of the circulation during T1; (b) the volume effect of generalized vasodilatation on CO by midazolam; (c) an additional effect of midazolam on resistance for venous return; (d) the volume effect of generalized vasodilatation on CO by fluid administration after midazolam. Cardiac output; *CVP* central venous pressure

To better elucidate the mechanism, the venous return curve of one representative rabbit was constructed from the average values obtained for right atrial pressure (a surrogate for central venous pressure) and cardiac output (Fig. 4), as previously described [18]. Three points in Fig. 4 represent the circulatory working points at T1(a), T2(c) and T3(d). The cardiac function curve did not change with increasing midazolam infusion rates (T1 and T2), but the working point left-shifted to lower values of CO and right atrial pressure. The Pmsf obtained from the venous return curve was also reduced. This Pmsf reduction may be explained by an increased vascular capacitance due to midazolam infusion, which shifted the stressed volume to the unstressed volume [19]. Vascular capacity is defined as the volume at a given pressure [19], assuming that the total intravascular volume in rabbits did not change. The recorded Pmsf reduction suggests an increase in vascular capacitance. Endotoxic rabbits with midazolam-induced haemodynamic changes were resuscitated at T3 until MAP was restored to baseline (i.e. before sedative use) to further test our hypothesis. Figure 3 shows that the C point returned to the D point, i.e. from the ascending curve to plateau status, after fluid infusion.

Augmented vascular capacitance and lower Pmsf reduced the venous return and therefore the SV and CO, despite attempts at compensation by increasing the HR. The CFI was not affected. The preload (i.e. GEDV) decreased significantly because of the reduced venous return, and the preload dependency (PPV) increased significantly. The Rvr decreased significantly from T0 to T2, which confirmed midazolam-induced venous dilatation and resulted in reduced preload and increased preload dependency.

Our study demonstrates that midazolam increases preload dependency in an endotoxic shock rabbit model. This result is inconsistent with a prior clinical observational study also conducted by our work team in which midazolam use did not increase the preload dependency in septic shock patients [20]. The following reasons may explain this inconsistency. First, the midazolam dose regimen in the prior study was a bolus dose of 2.5 mg and continuous infusion of 1.5 mg h, which is equivalent to the dose in the MID1 group in our study, and the effects on vascular tone were not obvious. Second, the sedation was titrated to Ramsay 3–4 points in the prior study, and the rabbits were anaesthetized using ketamine with midazolam. These sedatives are likely not comparable.

We recorded no differences in cardiac function as expressed by the CFI, i.e. the ratio of cardiac output to global end-diastolic volume. CFI correlates with left ventricular global systolic function [21, 22], and the recorded differences in SVI and CI cannot be attributable to an effect of acidosis on contractility, or on contractility itself, but to a preload midazolam effect.

Some limitations of the present study must be mentioned. First, we used SVV and PVV to reflect volume responsiveness. Previous studies demonstrated that SVV (directly measured using different pulse contour techniques or Doppler ultrasounds) or PPV reliably predicts the response to fluids when several prerequisites are met (e.g. absence of arrhythmias, tidal volume larger than 8 mL/kg, no respiratory movements) [23, 24]. These requirements were satisfied in the present study, and the use of SVV and PVV was likely reliable and effective.

Second, we used the end-inspiratory occlusion technique to draw the venous return curve for Pmsf computation [16]. Persichini et al. [14] recorded CO and CVP during end-inspiratory and end-expiratory ventilatory occlusions to describe a more precise curve. The description of this method was published after our study began, and our methods were chosen based on previously described literature.

In conclusion, midazolam affected the preload dependency at increasing doses in endotoxic shock rabbits undergoing norepinephrine infusion without affecting heart contractile function. These results suggest no major effects of midazolam on cardiac function in septic shock and that the haemodynamic fluctuations at large doses of midazolam were due to venous dilation. These data were derived from animal models, and further studies must be performed in humans to understand the possible interference of benzodiazepine in septic shocked patients.

## Conclusions

In conclusion, a high dose of midazolam administration in a septic shock model after fluid resuscitation and norepinephrine infusion increased the preload dependency via modification of vascular resistance. No effects on cardiac function were observed. Further studies must be performed in humans to understand the possible interference of sedative drugs on haemodynamics during septic shock.
